# Comparison of two front-of-pack nutrition labels for Brazilian consumers using a smartphone app in a real-world grocery store: A pilot randomized controlled study

**DOI:** 10.3389/fnut.2022.898021

**Published:** 2022-08-05

**Authors:** Alessandro Rangel Carolino Sales Silva, Cliona Ni Mhurchu, Lucilene Rezende Anastácio

**Affiliations:** ^1^Department of Food Science, Faculty of Pharmacy, Universidade Federal de Minas Gerais, Belo Horizonte, Brazil; ^2^National Institute for Health Innovation, University of Auckland, Auckland, New Zealand

**Keywords:** food labeling, nutritional labeling, mobile applications, front-of-pack nutrition labeling, warning labels

## Abstract

One of the suggestions for improving the understanding of food labels is implementing front-of-pack nutrition labeling (FoPNL), where nutritional information is objectively made available to consumers. Scientific data on the best FoPNL model to be adopted for the Brazilian population is still emerging, especially in real-world purchase situations. This study aims to evaluate/compare the proposed Brazilian and Mexican FoPNL systems, on different outcome measures, using an application, in dairy foods available in a supermarket aisle. This pilot randomized controlled trial in a real-world purchase situation was conducted in June/July 2021. A total of 230 participants were randomly allocated to one of the three study arms (Mexican and Brazilian FoPNL systems or control—nutritional information table and ingredients list). Using a smartphone, the participants scanned a product barcode and received the allocated FoPNL (with information about excessive added sugars, sodium, and/or saturated fat content) or the control. After, they answered questions related to our primary outcome (decision to buy or not to buy a product) and secondary outcomes (perceived healthiness, facilitation of a quick purchase decision, and identification of excess nutrients). The Mexican FoPNL system performed better in the primary outcome (3.74 ± 1.34) and “facilitation of a quick purchase decision” (3.59 ± 1.31), compared to the control (3.28 ± 1.45;*p* = 0.043 and 3.11 ± 1.42; *p* = 0.029). The Mexican FoPNL system performed better in supporting consumers to identify dairy foods, among the selected sample in this study, high in added sugars than the control (82.2% and 63.5% of correct answers, respectively; *p* = 0.009). For saturated fats, the Brazilian FoPNL resulted in 93.1% of correct answers against 48.2% for the control and 58.9% for the Mexican system (*p* ≤ 0.001). The Mexican FoPNL system facilitated consumer decision-making on when to buy or not to buy a selected dairy product and in helping to quickly decide which dairy products to buy, among the selected sample in this study, compared to the control. Considering the right answers of critical nutrients in excess or not, both models of FoPNL, delivered by a smartphone app, performed well.

## Introduction

Nutritional labeling aims to convey correct, precise, accurate, and conspicuous information to consumers and, consequently, influence their dietary decisions (1). However, most people do not correctly understand the complex information on food labels, such as the nutritional table and ingredients list (2–4). This problem contributes to reduced consumer interest in seeking information on labels and a preference for more salient information, such as nutritional claims, compromising the correct understanding of the nutritional profile of products (5–7).

Front-of-Pack Nutrition Labeling (FoPNL) has emerged as an alternative for better communication of nutrient content to consumers. The World Health Organization (WHO) recommends this strategy to aid healthier food choices since nutritional information would be displayed more clearly and encourage product reformulation (8). In some cases, the FoPNL provides clearer data on excessive nutrient content, such as calories, sugars, fats, and sodium (9).

Latin America has been leading the way regarding the FoPNL, with several countries opting to implement mandatory warning labels, especially the black octagon (Chile, Uruguay, and Mexico). For the implementation of FoPNL, it is also necessary that a nutritional profile model be implemented concurrently, serving as a parameter for identifying excessive nutrients in the food. The Mexican FoPNL label is a black octagon with the words “excess in” and uses a nutritional profile model with parameters for calories, sugars, saturated fats, trans fat, and sodium (10), based on the Pan-American Health Organization (PAHO) nutrient profile model (11). This Mexican system also includes a black box notice for the presence of caffeine and sweeteners in the products and that consumption is not suitable for children. Brazil has been discussing the topic for some years, and in 2020 it published legislation that proposes the implementation of the black magnifying glass model, with the words “high in” and a nutrient profile model with parameters for added sugars, saturated fats, and sodium, from October of 2022 (Table 1) (12, 13).

**Table 1:** Different front-of-pack nutrition labels and control and their respective parameters for categorizing excess added sugars, saturated fats, and sodium.

	**Mexican FoPNL system**	**Brazilian FoPNL system**	**Control**
	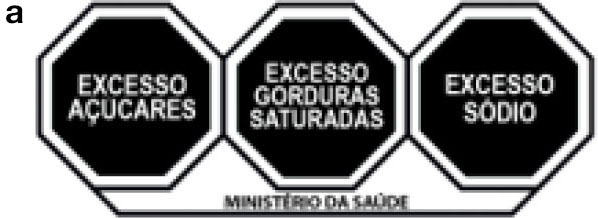	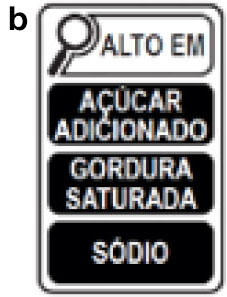	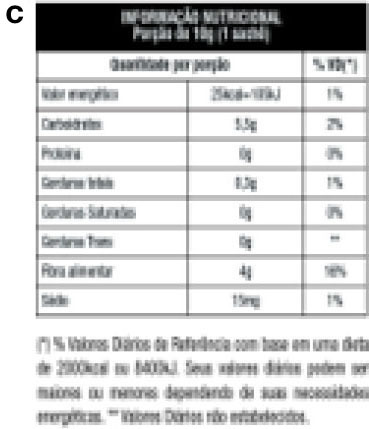
			and Ingredients List
Parameters for sodium	≥ 1 mg per 1 kcal or ≥ 300 mg	Solid food ≥ 600 mg per 100 g Liquid food ≥ 300 mg per 100 mL	NA
Parameters for saturated fats	≥ 10% of the total energy value (kcal)	Solid food ≥ 6 g per 100 g Liquid food ≥ 3 g per 100 mL	NA
Parameters for added sugars	≥ 10% of the total energy value (kcal)^a^	Solid food ≥ 15 g per 100 g Liquid food ≥ 7.5g per 100 mL	NA

NA, Not applicable.

a In the present study, we considered only added sugar instead of free sugar as Norma Oficial Mexicana regulates.

There is still no consensus on the best FoPNL label format and nutrient profile model, especially in a real-world purchase situation (in supermarkets, for example). A narrative review emphasizes the need for more studies in this setting (14). However, some labeling studies have used different strategies, such as the use of printed material on supermarket shelves (15), or a mobile application that provides nutritional information on-screen in the form of front-of-pack nutrition labeling for the consumer (16, 17).

The main objective of this study was to evaluate and compare the decision of to buy or not to buy a product (main outcome), perceived healthiness of selected products, facilitation of a quick purchase decision, and correct identification of excess nutrients of the Brazilian and Mexican FoPNL systems, through the use of an application for smartphones in a real-world purchase situation. The main hypothesis of our study is that the presence of FoPNL will have a positive impact on the evaluated outcomes, improving the understanding of healthiness, then the judgment and purchase decision, when compared to control.

## Materials and methods

### Trial design

The study was a randomized controlled trial with three-arms [Mexican FoPNL system, Brazilian FoPNL system, and control (nutritional information table and ingredients list)] delivered by a smartphone app and was approved by the Ethics Committee for Research with Human Beings of the Federal University of Minas Gerais, under protocol number 3.059.967.

### Participants

The inclusion criteria consisted of agreeing to participate in the study; being over 18 years old; and owning a Smartphone. The exclusion criteria were professionals in the field of food and nutrition. All participants consented by signing the Informed Consent Form.

### Interventions

#### Development of the RotulApp smartphone application and operation for the pilot study

The application consists of a smartphone platform available for free download (RotulApp, available on PlayStore for Android). Once installed, the participant completed their registration by answering a socioeconomic questionnaire on age, gender, education, income, profession, and marital status. Also, at the time of registration, the participant was randomized to one of the three arms of the study [Mexican FoPNL system ^a^ (black octagon, PAHO based nutritional profile model and “excess” descriptor), Brazilian FoPNL system ^b^ (magnifying glass, Brazilian Health Regulatory Agency (ANVISA) nutritional profile model and “high in” descriptor) and control ^c^ (nutritional information table and ingredients list, mandatory information displayed on foods in Brazil)] (Table 1).

RotulApp has a scanner system in which the participant when scanning the barcode of the product received one of the two front label models, according to randomization, or the control on his smartphone screen. The FoPNL models provided information on excessive levels of added sugars, saturated fats, and sodium, following previously determined parameters, as shown in Table 1.

By opening the application, the participant answered whether or not the presence at a grocery store and as soon as scanned and received a FoPNL model or the control, answered a brief questionnaire.

#### RotulApp database registration

The seven focus dairy food groups for the pilot study were: dairy drinks, milk curds (a fermented milk product made from warm milk and a bacterial yogurt starter), cream cheeses, yogurts, fermented kinds of milk, “petit Suisse” cheeses, and dairy desserts (n = 238 products). Brand-specific data for these products were collected in one supermarket in Belo Horizonte/MG in April of 2021. The collected products were positioned in a single aisle of the establishment and were chosen because national studies indicated that the labels of such products are the most consulted by consumers (18, 19).

The following product information was collected and incorporated into a product database: barcodes, the image of the front label of the products, and the image of the nutritional table and ingredients list. Data collection was performed using the Epicollect 5 software *via* smartphone, and the data were stored in the cloud. Subsequently, data were tabulated in Microsoft Excel version 2016 to classify each food according to the nutritional profile model of the allocated FoPNLs (Table 1). After that, results were incorporated into the RotulAPP application system, allowing the information in FoPNL format to be made available on the smartphone screen when scanning the products.

In Brazil, information on the content of sugars in foods is not mandatory on product labels, and only 49 of the 238 products in the study had this declaration. Therefore, we estimated the content of total sugars, adapting the method described by Scapin et al. (20), and then determined the added sugar content using the method proposed by the Pan American Health Organization (PAHO) (11).

#### Data collection

The application was available on the PlayStore for Android smartphones. The advertisement of the study was carried out through the creation of RotulApp's social media (Facebook and Instagram), in addition to flyers with information about the application, which were distributed in the supermarket, in Belo Horizonte, Minas Gerais, Brazil.

Recruitment (from June to July 2021) was undertaken online and in-store at a local supermarket closer to the University, where consumers were approached at the time of purchase (in a specific aisle of the establishment, with the registered products) and were invited to participate at the study. Participants who downloaded the application on their own cell phone received, on first use, a short online tutorial informing how and what types of products to scan. Participants who participated in the in-store data collection received instructions through an information flyer and the help of trained assistants. Overall, they were asked to scan the barcode of at least one product from the seven selected dairy food groups and then answer the questionnaire.

Two different randomization procedures were used, one at the time of the participant's registration on the platform (using their own smartphones) and the other using the Microsoft Excel version 2016 program for the in-store data collection participants, who used a specific smartphone, provided by the researchers, to data collection.

### Outcomes

The primary outcome of this study was the decision of whether to buy or not to buy a product and the secondary outcomes were the perceived healthiness of selected products, facilitation of a quick purchase decision, and the identification of excess nutrients. Likert scales and 5-point scales were used to establish a value for each response, always in ascending order: “*Does this nutritional labeling model helps me decide when to buy or not buy a product?*” (Outcome: the decision of to buy or not to buy a product) and “*Does this nutritional labeling model help me quickly decide which products to buy?”* (Outcome: facilitation of a quick purchase decision) (*Strongly disagree* = *1; Partially disagree* = *2; Neither agree nor disagree* = *3; Partially agree* = *4; Totally agree* = *5*). For the outcome of perceived healthiness, we used the question “*Is this product considered healthy?” (Not healthy* = *1; Unhealthy* = *2; Healthy* = *3; Very healthy* = *4; Extremely healthy* = *5)*.

In assessing understanding of the critical nutritional content of the products—“*In your opinion, does the scanned product contain any excessive nutrient or any substance that could harm a healthy diet?,”* 1 point was allocated for each marked nutrient option that the consumer believes to be in excess in the product and 0 for the unmarked option. 1 point was also allocated for any nutrient in excess and 0 when the opposite, in two different situations: situation 1—primary approach: only applying PAHO's nutritional profile model for the three arms; situation 2—secondary approach: applying the nutritional profile model of ANVISA for the Brazilian FoPNL system, the nutritional profile model implemented in Mexico for the Mexican FoPNL system, and the nutritional profile model from PAHO for control (Table 2).

**Table 2:** Differences between the three nutritional profile models (NPM) used in assessing understanding of the critical nutritional content of the products.

	**Mexican NPM**	**Brazilian NPM**	**PAHO NPM**
Parameters for sodium	≥ 1 mg per 1 kcal or ≥ 300 mg	Solid food ≥ 600 mg per 100 g Liquid food ≥ 300 mg per 100 mL	≥ 1 mg per 1 kcal
Parameters for saturated fats	≥ 10% of the total energy value (kcal)	Solid food ≥ 6 g per 100 g Liquid food ≥ 3 g per 100 mL	≥ 10% of the total energy value (kcal)
Parameters for added sugars	≥ 10% of the total energy value (kcal)*	Solid food ≥ 15 g per 100 g Liquid food ≥ 7.5 g per 100 mL	≥ 10% of the total energy value (kcal)*

* In the present study, we considered only added sugar instead of free sugar as Norma Oficial Mexicana regulates.

After that, a sum was made between the value of the response provided by the participant and the value categorized by the nutritional profile model, obtaining three possible values, 0 and 2, signaling the right answer (in the presence or absence of excessive nutrients) and 1 signaling the wrong answer. We also evaluated the ability of FoPNL models delivered by a smartphone app to support consumers to detect products with high content of critical nutrients. In all cases, only the assessments of added sugars, saturated fats, and sodium were analyzed. The same outcomes were also analyzed considering only products eligible for the Brazilian FoPNL system and presented as Supplementary Data.

### Statistical methods

Differences between FoPNL models and control, delivered by a smartphone app, were statistically compared for all study outcomes. The results were statistically treated by analysis of variance (ANOVA) with *post-hoc* Tukey test (continuous variables) or Pearson's chi-square test or Fisher's exact test (categorical variables), considering p ≤ 0.05 as significant. The normality of continuous data was verified using the Kolmogorov-Smirnov test. The statistical software IBM SPSS (Statistics for Windows, Version 20.0. Armonk, NY: IBM Corp) was used.

## Results

**Figure 1:**
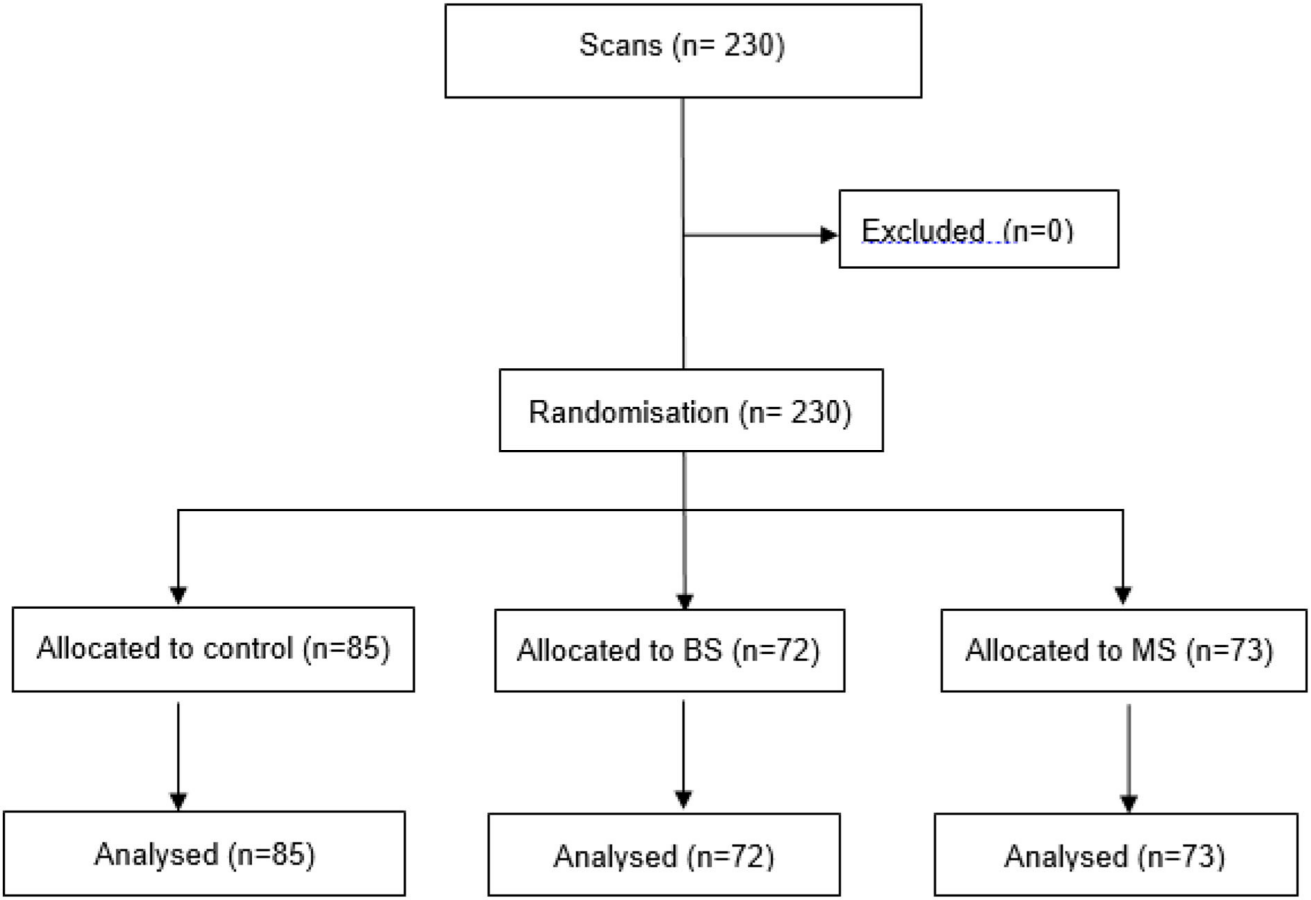
CONSORT diagram of scans through the trial. Brazilian System (BS), Mexican System (MS).

Information was recorded for 238 available dairy products, divided into seven categories, with the yogurt category predominating with 153 products (64% of all dairy products). There were 230 scans of registered products (Figure 1).

The most scanned category was yogurt (n = 139 scans, 60% total scans), being also the most scanned in the three different arms of the study. Dairy desserts had the highest average content of added sugars, saturated fats, and sodium per 100 g (15.3 ± 3.0 g, 2.9 ± 0.4 g, and 98.3 ± 56.1 mg, respectively) (Tables 3, 4).

**Table 3:** Number of registered products for the pilot study (*n* = 238), number and percentage of scanned products (*n* = 230), grouped by category, and mean and SD of the evaluated nutrients (per 100 g or 100 ml).

**Registered products by category (n)**	**Total number of scans (%)**	**Added sugars (g)**	**Saturated fats (g)**	**Sodium (mg)**
		**Mean (Standard deviation)**		
Cream cheeses (3)	1 (0.5)	4.0 (NA)	2.6 (NA)	27.0 (NA)
Curds (3)	2 (0.9)	0.0 (0.0)	0.0 (0.0)	73.1 (0.0)
Dairy drinks (31)	12 (5.2)	7.8 (0.7)	0.8 (0.3)	41.1 (12.2)
Dairy desserts (6)	12 (5.2)	15.3 (3.0)	2.9 (0.4)	98.3 (56.1)
“Petit Suisse” cheeses (11)	14 (6.1)	5.3 (1.0)	2.0 (0.3)	66.1 (5.7)
Fermented milks (31)	50 (21.7)	6.3 (1.6)	0.4 (0.6)	38.7 (14.9)
Yogurts (153)	139 (60.4)	3.8 (2.8)	1.4 (1.1)	49.0 (11.9)
TOTAL (238)	230 (100.0)			

NA, Not applicable.

**Table 4:** Number and percentage of scans, grouped by category and study arm.

**Category**	**Control (*n* = 85)**	**BS (*n* = 72)**	**MS (*n* = 73)**	**Total (*n* = 230)**	***P*-value**
	***N*** **(% of products within the study arm)**				
Cream cheeses	0 (0.0)	0 (0.0)	1 (1.4)	1	0.091
Curds	1 (1.2)	0 (0.0)	1 (1.4)	2	
Dairy drinks	3 (3.5)	4 (5.5)	5 (6.8)	12	
Dairy desserts	1 (1.2)	9 (12.5)	2 (2.7)	12	
“Petit Suisse” cheeses	4 (4.7)	7 (9.7)	3 (4.1)	14	
Fermented milks	21 (24.7)	12 (16.7)	17 (23.3)	50	
Yogurts	55 (64.7)	40 (55.6)	44 (60.3)	139	
n and % of eligible products for FoPNL	NA	19 (26.4)	73 (100.0)	NA	NA

NA, Not applicable; BS, Brazilian FoPNL System; MS, Mexican FoPNL System.

Pearson Chi-Square test.

Table 3 also indicates the percentage of dairy products categorized as having an excess of at least one critical nutrient in the Brazilian FoPNL system and Mexican FoPNL system (26.4% and 100%, respectively). As a result, for the Brazilian FoPNL system, 73.6% of the eligible products for scanning did not display any warning information. The general characteristics of the participants in RotulApp (n = 230) during the data collection period are described in Table 5.

**Table 5:** General characteristics and mean and SD of the age of participants in the RotulApp application, grouped by study arm.

**General characteristics**	**Total (*n* = 230)**	**Control (*n* = 85)**	**BS (*n* = 72)**	**MS (*n* = 73)**	**P-value**
Sex					
Male	87 (37.8%)	29 (34.1%)	24 (33.3%)	34 (46.6%)	0.175
Female	143 (62.2%)	56 (65.9%)	48 (66.7%)	39 (53.4%)	
Education					
First to eighth grade (complete and incomplete)	7 (3.0%)	1 (1.2%)^a^	2 (2.8%)^a^	4 (5.5%)^a^	0.003*
High school (complete and incomplete)	47 (20.4%)	14 (16.5%)^a^	13 (18.1%)^a^	20 (27.4%)^a^	
Graduation (complete and incomplete)	117 (50.9%)	50 (58.8%)^b^	45 (62.5%)^b^	22 (30.1%)^a^	
Postgraduate	59 (25.7%)	20 (23.5%)^ab^	12 (16.7%)^b^	27 (37.0%)^a^	
State					
Espírito Santo	1 (0.4%)	0 (0.0%)	0 (0.0%)	1 (1.4%)	0.425
Minas Gerais	228 (99.1%)	84 (98.8%)	72 (100.0%)	72 (98.6%)	
São Paulo	1 (0.4%)	1 (1.2%)	0 (0.0%)	0 (0.0%)	
Responsible for shopping?					
Yes	176 (76.5%)	67 (78.8%)^a^	44 (61.1%)^b^	65 (89.0%)^a^	<0.001*
No	54 (23.5%)	18 (21.2%)^a^	28 (38.9%)^b^	8 (11.0%)^a^	
Consume any study product?					
Yes	219 (95.2%)	82 (96.5%)	70 (97.2%)	67 (91.8%)	0.244
No	11 (4.8%)	3 (3.5%)	2 (2.8%)	6 (8.2%)	
**Numerical parameters**	**Total (*****n*** **=** **230)**	**Control (*****n*** **=** **85)**	**BS (*****n*** **=** **72)**	**MS (*****n*** **=** **73)**	
Age [Average (Standard Deviation)]	35.3 (11.2)	35.7 (10.8)	33.2 (10.6)	36.8 (12.1)	0.199

BS, Brazilian FoPNL System; MS, Mexican FoPNL System.

* P < 0.05—two-sided; different letters mean P < 0.05.

Pearson Chi-Square test.

When analyzing right and wrong answers about nutrients, in relation to the identification of excessive added sugars, in situation 1 (PAHO's nutritional profile model for the three arms), the Mexican FoPNL system outperformed the control (82.2% of right answers against 63.5%). In situation 2 (specific nutritional profile model for each arm), the Mexican FoPNL system outperformed both the Brazilian FoPNL system and control (82.2% of right answers against 65.3 and 63.5%, respectively).

As for the identification of excessive saturated fats, in situation 1, the Mexican FoPNL system outperformed the Brazilian FoPNL system (58.9% of right answers against 38.9%). In situation 2, the Brazilian FoPNL system stood out with 93.1% of correct answers against 58.9% of correct answers in the Mexican FoPNL system group and 48.2% in the control group (Table 6).

**Table 6:** Right and wrong answers about nutrient content, by study arm, considering different nutritional profile models.

**In your opinion, does the scanned product contain any excessive nutrients or any substance that could harm a healthy diet?**									
	**Control**	**BS**	***P*** **-value**	**Control**	**MS**	***P*** **-value**	**BS**	**MS**	**P-value**
	**(*****n*** = **85)**	**(*****n*** = **72)**		**(*****n*** = **85)**	**(*****n*** = **73)**		**(*****n*** = **72)**	**(*****n*** = **73)**	
**Situation 1) NP: OPAS for control, MS, and BS**									
Sugars									
Right	54 (63.5%)	50 (69.4%)	0.435	54 (63.5%)	60 (82.2%)	0.009*	50 (69.4%)	60 (82.2%)	0.073
Wrong	31 (36.5%)	22 (30.6%)		31 (36.5%)	13 (17.8%)		22 (30.6%)	13 (17.8%)	
Saturated fats									
Right	41 (48.2%)	28 (38.9%)	0.240	41 (48.2%)	43 (58.9%)	0.180	28 (38.9%)	43 (58.9%)	0.016*
Wrong	44 (51.8%)	44 (61.1%)		44 (51.8%)	30 (41.1%)		44 (61.1%)	30 (41.1%)	
Sodium									
Right	60 (70.6%)	53 (73.6%)	0.674	60 (70.6%)	55 (75.3%)	0.503	53 (73.6%)	55 (75.3%)	0.811
Wrong	25 (29.4%)	19 (26.4%)		25 (29.4%)	18 (24.7%)		19 (26.4%)	18 (24.7%)	
**Situation 2) NP: ANVISA for BS, Mexican for MS, and OPAS for control**									
Sugars									
Right	54 (63.5%)	47 (65.3%)	0.820	54 (63.5%)	60 (82.2%)	0.009*	47 (65.3%)	60 (82.2%)	0.021*
Wrong	31 (36.5%)	25 (34.7%)		31 (36.5%)	13 (17.8%)		25 (34.7%)	13 (17.8%)	
Saturated fats									
Right	41 (48.2%)	67 (93.1%)	<0.001*	41 (48.2%)	43 (58.9%)	0.180	67 (93.1%)	43 (58.9%)	<0.001*
Wrong	44 (51.8%)	5 (6.9%)		44 (51.8%)	30 (41.1%)		5 (6.9%)	30 (41.1%)	
Sodium									
Right	60 (70.6%)	58 (80.6%)	0.150	60 (70.6%)	55 (75.3%)	0.503	58 (80.6%)	55 (75.3%)	0.449
Wrong	25 (29.4%)	14 (19.4%)		25 (29.4%)	18 (24.7%)		14 (19.4%)	18 (24.7%)	

* P < 0.05—two-sided. NP, Nutritional profile; BS, Brazilian FoPNL System; MS, Mexican FoPNL System.

Pearson Chi-Square test.

Analyzing the ability of the FoPNL systems to make the consumers identify only the excess of critical nutrients, the Mexican FoPNL system performed better than control and the Brazilian FoPNL system, in situation 1, and the Brazilian and Mexican FoPNL systems performed better than control in situation 2 (Table 7).

**Table 7:** Right and wrong answers about excessive nutrients, by study arm, considering different nutritional profile models.

**In your opinion, does the scanned product contain any excessive nutrients or any substance that could harm a healthy diet?**									
**Situation 1) NP: OPAS for control, MS, and BS**									
Sugars	Control (*n =* 68)	BS (*n =* 59)	*P-*value	Control (*n =* 68)	MS (*n =* 56)	*P-*value	BS (*n =* 59)	MS (*n =* 56)	*P-*value
Right	39 (57.4%)	37 (62.7%)	0.539	39 (57.4%)	44 (78.6%)	0.012*	37 (62.7%)	44 (78.6%)	0.062
Wrong	29 (42.6%)	22 (37.3%)		29 (42.6%)	12 (21.4%)		22 (37.3%)	12 (21.4%)	
Saturated fats	Control (*n =* 45)	BS (*n =* 50)	*P-*value	Control (*n =* 45)	MS (*n =* 46)	*P-*value	BS (*n =* 50)	MS (*n =* 46)	*P-*value
Right	6 (13.3%)	6 (12.0%)	0.845	6 (13.3%)	17 (37.0%)	0.010*	6 (12.0%)	17 (37.0%)	0.004*
Wrong	39 (86.7%)	44 (88.0%)		39 (86.7%)	29 (63.0%)		44 (88.0%)	29 (63.0%)	
Sodium	Control (*n =* 14)	BS (*n =* 7)	*P-*value	Control (*n =* 14)	MS (*n =* 13)	*P-*value	BS (*n =* 7)	MS (*n =* 13)	*P-*value
Right	7 (50.0%)	1 (14.3%)	0.112	7 (50.0%)	9 (69.2%)	0.310	1 (14.3%)	9 (69.2%)	0.019*
Wrong	7 (50.0%)	6 (85.7%)		7 (50.0%)	4 (30.8%)		6 (85.7%)	4 (30.8%)	
**Situation 2) NP: ANVISA for BS, Mexican for MS, and OPAS for control**									
Sugars	Control (*n =* 68)	BS (*n =* 18)	*P-*value	Control (*n =* 68)	MS (*n =* 56)	*P-*value	BS (*n =* 18)	MS (*n =* 56)	*P-*value
Right	39 (57.4%)	15 (83.3%)	0.043*	39 (57.4%)	44 (78.6%)	0.012*	15 (83.3%)	44 (78.6%)	0.662
Wrong	29 (42.6%)	3 (16.7%)		29 (42.6%)	12 (21.4%)		3 (16.7%)	12 (21.4%)	
Saturated fats	Control (*n =* 45)	BS (*n =* 1)	*P-*value	Control (*n =* 45)	MS (*n =* 46)	*P-*value	BS (*n =* 1)	MS (*n =* 46)	*P-*value
Right	6 (13.3%)	1 (100.0%)	0.152	6 (13.3%)	17 (37.0%)	0.010*	1 (100.0%)	17 (37.0%)	0.383
Wrong	39 (86.7%)	0 (0.0%)		39 (86.7%)	29 (63.0%)		0 (0.0%)	29 (63.0%)	
Sodium	Control (*n =* 14)	BS (*n =* 0)	*P-*value	Control (*n =* 14)	MS (*n =* 13)	*P-*value	BS (*n =* 0)	MS (*n =* 13)	*P-*value
Right	NA	NA	NA	7 (50.0%)	9 (69.2%)	0.310	NA	NA	NA
Wrong	NA	NA		7 (50.0%)	4 (30.8%)		NA	NA	

* P < 0.05—two-sided. NP, Nutritional profile; BS, Brazilian FoPNL System; MS, Mexican FoPNL System.

Pearson Chi-Square and Fisher's Exact Test.

There was a statistically significant difference (*p* ≤ 0.05) between the Mexican FoPNL system and both the control and the Brazilian FoPNL system, for the outcomes “facilitation of a quick purchase decision” (3.59 ± 1.31 against 3.11 ± 1.42 (*p* = 0.029) and 3.07 ± 1.53 (*p* = 0.030), respectively) and “decision of to buy or not to buy a product” (3.74 ± 1.34 against 3.28 ± 1.45 (*p* = 0.043) and 3.10 ± 1.59 (*p* = 0.009), respectively) (Table 8). Considering only products that were scanned and eligible for the Brazilian FoPNL (*n* = 19), this system outperformed both the control and Mexican FoPNL systems for the outcome of “perceived healthiness” (2.26 ± 1.45 against 3.24 ± 1.29 and 3.18 ± 1.25, respectively) (Supplementary Data 2).

**Table 8:** Mean, median, and SD of the scale values, according to the different models of the study, for perceived healthiness of selected products, facilitation of a quick purchase decision, and decision of to buy or not to buy a product, based on the results obtained with the RotulApp application.

	**Control (*n =* 85)**	**BS (*n =* 2)**	***P-*value**	**Control (*n =* 85)**	**MS (*n =* 73)**	***P-*value**	**BS (*n =* 72)**	**MS (*n =* 73)**	**P–value**
**Is this product considered healthy?**									
Mean ± standard deviation	3.24 ± 1.29	3.10 ± 1.47	0.531	3.24 ± 1.29	3.18 ± 1.25	0.778	3.10 ± 1.47	3.18 ± 1.25	0.721
Median (interquartile range)	4.00 (2.00–4.00)	3.50 (2.00–4.00)		4.00 (2.00–4.00)	4.00 (2.00–4.00)		3.50 (2.00–4.00)	4.00 (2.00–4.00)	
**Does this nutritional labeling model help me quickly decide which products to buy?**									
Mean ± standard deviation	3.11 ± 1.42	3.07 ± 1.53	0.878	3.11 ± 1.42^b^	3.59 ± 1.31^a^	0.029*	3.07 ± 1.53^b^	3.59 ± 1.31^a^	0.030*
Median (interquartile range)	3.00 (2.00–4.00)	3.00 (1.00–4.00)		3.00 (2.00–4.00)	4.00 (2.50–5.00)		3.00 (1.00–4.00)	4.00 (2.50–5.00)	
**Does this nutritional labeling model help me decide when to buy or not buy a product?**									
Mean ± standard deviation	3.28 ± 1.45	3.10 ± 1.59	0.447	3.28 ± 1.45^b^	3.74 ± 1.34^a^	0.043*	3.10 ± 1.59^b^	3.74 ± 1.34^a^	0.009*
Median (interquartile range)	4.00 (2.00–5.00)	3.00 (1.00–5.00)		4.00 (2.00–5.00)	4.00 (2.50–5.00)		3.00 (1.00–5.00)	4.00 (2.50–5.00)	

* P < 0.05—two-sided; different letters mean P < 0.05. B, Brazilian FoPNL System; MS, Mexican FoPNL System.

ANOVA (post-hoc Tukey test).

## Discussion

In the present study, there was a statistically significant difference between the Mexican FoPNL system and both the control and the Brazilian FoPNL system, all delivered by a smartphone app, for the outcome “decision of to buy or not to buy a product” amongst dairy foods by the sample of this study. The Mexican FoPNL system presented a significantly higher value than the control and the Brazilian FoPNL system. The closer to 5, the greater the agreement of consumers that the model assisted in the decision of whether to buy a product or not, but the worse performance of the Brazilian FoPNL system may be explained by the fact that in 73.6% of the scans of this arm, no additional warning information was displayed to the consumer, due to the relatively permissive nutritional profile model for this system. The same difference was indicated between the Mexican FoPNL system and both the control and the Brazilian FoPNL system, for the outcome “facilitation of a quick purchase decision.”

Although the present study did not show a statistically significant difference between the models for the outcome of “perceived healthiness,” results from an Uruguayan online study indicated that FoPNL favored healthier food choices compared to the control (21). However, a recent systematic review indicated that, based on a limited number of studies in a real purchase situation, the influence of FoPNL on the purchase of healthier products was small (14).

As noted above, although the number of scans was proportional for each arm of the study, the percentage of products that received warning information in the Brazilian FoPNL system arm was only 26.4% (*n* = 19) (Supplementary Data). This is due to the nutritional profile model recommended for this FoPNL, which is a more permissive profile compared to those used in other Latin American countries (22, 23) and models proposed by the Brazilian regulatory agency and PAHO, it is, in fact, a model that categorizes fewer foods as containing excessive nutrients (11, 24).

Considering only products that displayed the Brazilian FoPNL, there was a statistically significant difference for the outcome “perceived healthiness,” where the perception of healthiness was substantially reduced when compared to the control and the Mexican FoPNL. This result suggests that the Brazilian FoPNL reduces the perception of healthiness of dairy products, among the selected sample in this study, with critical nutrients in excess, as long as the model is present on the food label (Supplementary Data).

In the regulatory implementation of the Brazilian FoPNL system with the nutritional profile model proposed by ANVISA, the possible absence of the magnifying glass symbol (due to a more permissive nutritional profile model) will mean that the consumer will only have access to information already provided by the nutritional table and ingredients list of the product and will potentially believe that products without FoPNL are healthier than before the implementation of the regulation. A Chilean study, with the first-year evaluation of FoPNL implementation, indicated increased consumption of products without FoPNL, even in the same category of products that normally received a labeling warning (25).

Regarding the identification of excessive nutrients, considering the nutritional profile models proposed for each FoPNL delivered by a smartphone app (situation 2), there was a statistically significant difference between the right answers of excess or not added sugars [the Mexican FoPNL system stood out with the highest percentage of correct answers (82.2% against 63.5% of the control and 65.3% of the Brazilian FoPNL system)] and saturated fats (the Brazilian FoPNL system stood out with the highest percentage of correct answers (93.1% against 48.2% of the control and 58.9% of the Mexican FoPNL system)]. The differences were also observed when just the ability to identify the excess of critical nutrients was evaluated. It is noteworthy that for saturated fats, in the Brazilian FoPNL system arm, only one product out of the 72 scanned products showed an excess of this nutrient.

These findings show that the presence of FoPNL favors the better interpretation of the nutritional content of the product, a finding also seen in other studies (9, 14, 17). An online study carried out in 2021 with 2400 Brazilian participants also indicated that the use of different FoPNL models, including the magnifying glass and the octagon, both increased the understanding of the nutritional content of products, but this studies evaluated the effectiveness of the design only and not the concomitant application of nutritional profile models (26).

In the case of added sugars in situation 1, no statistically significant difference was found between the two models of FoPNL in identifying or not this nutrient was in excess (difference was only found when comparing the Mexican FoPNL system with the control group (82.2% of right answers against 63.5%). When considering only the identification of excessive nutrients in selected dairy products, both FoPNL systems performed better than the control, in situation 2. A similar finding to the study by Deliza et al. (27), which pointed out that different FoPNL models, such as warnings and the magnifying glass, seem to be more appropriate for this purpose, but also without significant difference between the models. In that study, the models were different in design, but not the nutritional profile (27).

In both situations (applying the nutritional profile models proposed for each FoPNL model and considering PAHO's nutritional profile model as a reference for all arms), consumers exposed only to the nutritional table, and ingredients list demonstrated less understanding of the sugar content of the products, demonstrated by the lowest number of correct answers. In general, FoPNL effectively informs and indicates high levels of critical nutrients and encourages healthier behaviors, especially considering that supermarket purchases are usually made in a rush and limited time (28–30). A meta-analysis of experimental studies found that FoPNL warnings lowered the sodium and sugar content of consumer purchases compared with no labels (9).

We can mention as strengths of this work the remote intervention through an application, which allows changes to the study design without harming its progress and simulates the implementation of FoPNL delivered by a smartphone app in a real purchase situation. In Brazil, there are no previous studies that developed applications for smartphones within the scope of FoPNL and on the effect of this strategy in a real purchase situation, which makes this work novel. Fuchs et al. indicate that the development of a tailoring framework for the personalization of digital food labels represents a promising purchase-intervention even in the absence of FoPNL and the advantage of this label customization through mobile apps over standardized labels is the real-time presentation of individualized and customized labels based on different nutritional profiles (31).

In addition, discussions for the implementation of FoPNL must have a broad approach, with the active participation of all regulatory and public policy spheres, accompanied by education and dissemination measures, as well as surveillance and sanctions in case of non-compliance (32). In this study, we evaluated not only the FoPNL designs but also the closest to what the corresponding legislation describes for descriptors and nutritional profile models.

As limitations, we highlight that despite the performed randomization for the label's groups, the chi-square test indicated that for the characteristics “education” and “responsible for shopping,” there was a significant difference between the study arms, with a predominance of participants with incomplete graduation in the Brazilian FoPNL system arm and with a postgraduate education in the Mexican FoPNL system arm, in addition to a smaller number of participants responsible for purchases also in the Brazilian FoPNL system arm. The use of two different randomization lists could be responsible for these imbalances. Also, the voluntary use of an application for FoPNL may have included participants motivated to find and use nutritional information. Although, we believe that the main motivation of the participants was related to the contribution to the research of the local university. We can not disregard the possibility of a social desirability bias with some of the questions used. Also, our study tests the use of the app in a specific aisle with predetermined products, the results may differ in other food groups.

The use of FoPNL through an application is also a weakness considering that this is not how shoppers usually encounter and use labels in-store. Testing understanding of FoPNL when used on a smartphone to scan food products, involves different cognitive processes than what is expected when using FoPNL under real-life conditions. The low number of participants could also be a limiting factor for general conclusions, as it was a convenience sample and this was a pilot study. However, considering the significance level of 5%, the study sample had a power of 83.2% to demonstrate the mean difference in the decision to buy or not to buy a product (our primary outcome) between the Mexican (3.74 ± 1.34) and the Brazilian (3.10 ± 1.59) FoPNL systems.

The obtained results reinforce the need to adopt a FoPNL model in food regulatory legislation since the current labeling makes it difficult for consumers to understand the information, and FoPNL is, therefore, an important modification strategy, given the benefits it provides to consumers (29, 33).

## Conclusion

The Mexican FoPNL system delivered by a smartphone app supported consumer decision-making regarding whether to buy or not to buy a product amongst dairy foods and in helping to quickly decide which dairy products to buy, compared to the control and among the selected sample in this study. With regards to correctly identifying critical nutrients in excess or not, both models of FoPNL performed well, with the Mexican FoPNL system delivered by a smartphone app standing out for added sugars and saturated fats (only using PAHO's nutritional profile model for the three arms) and the Brazilian FoPNL system delivered by a smartphone app standing out for saturated fats (considering the nutritional profile models proposed for each FoPNL), also among the selected dairy samples in this study.

## Data availability statement

The raw data supporting the conclusions of this article will be made available by the authors, without undue reservation.

## Ethics statement

The studies involving human participants were reviewed and approved by Universidade Federal de Minas Gerais Ethics Committee (01919218.1.0000.5149). The patients/participants provided their written informed consent to participate in this study.

## Author contributions

AS: data collection, project management, methodology, writing—preparation of the original manuscript, writing— proofreading and editing, and statistical analysis. LA: supervision, methodology, writing—proofreading and editing, and statistical analysis. CN: methodology—feedback on study design and writing—proofreading and editing. All authors contributed to the article and approved the submitted version.

## Funding

This study was funded by Coordenação de Aperfeiçoamento de Pessoal de Nível Superior (CAPES), Conselho Nacional de Desenvolvimento Científico e Tecnológico—CNPq/Ministério da Saúde do Brasil (442990/2019-7), Fundação de Amparo à Pesquisa do Estado de Minas Gerais—FAPEMIG (APQ-00341-21), and Pró-Reitoria de Pesquisa da Universidade Federal de Minas Gerais.

## Conflict of interest

The authors declare that the research was conducted in the absence of any commercial or financial relationships that could be construed as a potential conflict of interest.

## Publisher's note

All claims expressed in this article are solely those of the authors and do not necessarily represent those of their affiliated organizations, or those of the publisher, the editors and the reviewers. Any product that may be evaluated in this article, or claim that may be made by its manufacturer, is not guaranteed or endorsed by the publisher.
