# Corrigendum: Comparison of two front-of-pack nutrition labels for Brazilian consumers using a smartphone app in a real-world grocery store: A pilot randomized controlled study

**DOI:** 10.3389/fnut.2022.1040011

**Published:** 2022-10-13

**Authors:** Alessandro Rangel Carolino Sales Silva, Cliona Ni Mhurchu, Lucilene Rezende Anastácio

**Affiliations:** ^1^Department of Food Science, Faculty of Pharmacy, Universidade Federal de Minas Gerais, Belo Horizonte, Brazil; ^2^National Institute for Health Innovation, University of Auckland, Auckland, New Zealand

**Keywords:** food labeling, nutritional labeling, mobile applications, front-of-pack nutrition labeling, warning labels

In the published article, there was an error in the Funding statement. Coordination for the Improvement of Higher Education Personnel (CAPES), and Conselho Nacional de Desenvolvimento Científico e Tecnológico-CNPq and Ministério da Saúde-MS (308771/2020-6) should be removed. The correct Funding statement appears below.

## Funding

This study was funded by Coordenação de Aperfeiçoamento de Pessoal de Nível Superior (CAPES), Conselho Nacional de Desenvolvimento Científico e Tecnológico—CNPq/Ministério da Saúde do Brasil (442990/2019-7), Fundação de Amparo à Pesquisa do Estado de Minas Gerais—FAPEMIG (APQ-00341-21), and Pró-Reitoria de Pesquisa da Universidade Federal de Minas Gerais.

The authors apologize for this error and state that this does not change the scientific conclusions of the article in any way.

The original article has been updated.

## Publisher's note

All claims expressed in this article are solely those of the authors and do not necessarily represent those of their affiliated organizations, or those of the publisher, the editors and the reviewers. Any product that may be evaluated in this article, or claim that may be made by its manufacturer, is not guaranteed or endorsed by the publisher.

