# Supplementary material for: Comparison of two front-of-pack nutrition labels for Brazilian consumers using a smartphone app in a real-world grocery store: A pilot randomized controlled study
Source: Front Nutr. 2022 Aug 5;9:898021. doi: 10.3389/fnut.2022.898021 (PMC9389176; doi:10.3389/fnut.2022.898021)
Supplement: Supplementary file 1 [file Data_Sheet_1.docx]

Supplementary Material

**Supplementary data 1 –** Right and wrong answers about excessive nutrients, by study arm, considering different nutritional profile models and only products that received FoPNL for the Brazilian system.

| In your opinion, does the scanned product contain any excessive nutrients or any substance that could harm a healthy diet? | | | | | | | | |  |
| --- | --- | --- | --- | --- | --- | --- | --- | --- | --- |
| Situation 1) NP: OPAS for control, MS, and BS | | | | | | | | |  |
|  | **Control (n = 85)** | **BS (n = 19)** | **P value** | **Control (n = 85)** | **MS (n = 73)** | **P value** | **BS (n = 19)** | **MS (n = 73)** | **P value** |
| Sugars |  |  |  |  |  |  |  |  |  |
| Right | 54 (63.5%) | 15 (78.9%) | 0.199 | 54 (63.5%) | 60 (82.2%) | 0.009* | 15 (78.9%) | 60 (82.2%) | 0.746 |
| Wrong | 31 (36.5%) | 4 (21.1%) |  | 31 (36.5%) | 13 (17.8%) |  | 4 (21.1%) | 13 (17.8%) |  |
| Saturated fats |  |  |  |  |  |  |  |  |  |
| Right | 41 (48.2%) | 9 (47.4%) | 0.945 | 41 (48.2%) | 43 (58.9%) | 0.180 | 9 (47.4%) | 43 (58.9%) | 0.366 |
| Wrong | 44 (51.8%) | 10 (52.6%) |  | 44 (51.8%) | 30 (41.1%) |  | 10 (52.6%) | 30 (41.1%) |  |
| Sodium |  |  |  |  |  |  |  |  |  |
| Right | 60 (70.6%) | 14 (73.7%) | 0.788 | 60 (70.6%) | 55 (75.3%) | 0.503 | 14 (73.7%) | 55 (75.3%) | 0.882 |
| Wrong | 25 (29.4%) | 5 (26.3%) |  | 25 (29.4%) | 18 (24.7%) |  | 5 (26.3%) | 18 (24.7%) |  |
|  | **Situation 2)** NP: ANVISA for BS, Mexican for MS, and OPAS for control | | | | | | | | |
| Sugars |  |  |  |  |  |  |  |  |  |
| Right | 54 (63.5%) | 16 (84.2%) | 0.082 | 54 (63.5%) | 60 (82.2%) | 0.009* | 16 (84.2%) | 60 (82.2%) | 0.836 |
| Wrong | 31 (36.5%) | 3 (15.8%) |  | 31 (36.5%) | 13 (17.8%) |  | 3 (15.8%) | 13 (17.8%) |  |
| Saturated fats |  |  |  |  |  |  |  |  |  |
| Right | 41 (48.2%) | 19 (100.0%) | <0.001* | 41 (48.2%) | 43 (58.9%) | 0.180 | 19 (100.0%) | 43 (58.9%) | 0.001* |
| Wrong | 44 (51.8%) | 0 (0.0%) |  | 44 (51.8%) | 30 (41.1%) |  | 0 (0.0%) | 30 (41.1%) |  |
| Sodium |  |  |  |  |  |  |  |  |  |
| Right | 60 (70.6%) | 14 (73.7%) | 0.788 | 60 (70.6%) | 55 (75.3%) | 0.503 | 14 (73.7%) | 55 (75.3%) | 0.882 |
| Wrong | 25 (29.4%) | 5 (26.3%) |  | 25 (29.4%) | 18 (24.7%) |  | 5 (26.3%) | 18 (24.7%) |  |

* P < 0.05; different letters mean P<0.05 NP = Nutritional profile ; BS = Brazilian FoPNL System ; MS = Mexican FoPNL System

Pearson Chi-Square test

**Supplementary data 2 -** Mean, Median and Standard Deviation of the scale values, according to the different models of the study and only considering products that received FoPNL for the Brazilian system, for perceived healthiness of selected products, facilitation of a quick purchase decision and decision of to buy or not to buy a product, based on the results obtained with the RotulApp application

|  | **Control (n = 85)** | **BS (n = 19)** | **P value** | **Control (n = 85)** | **MS (n = 73)** | **P value** | **BS (n = 19)** | **MS (n = 73)** | **P value** |
| --- | --- | --- | --- | --- | --- | --- | --- | --- | --- |
| Is this product considered healthy? | | | | | | | | | |
| Mean ± Standard Deviation | 3.24±1.29 | 2.26±1.45 | 0.004* | 3.24±1.29 | 3.18±1.25 | 0.778 | 2.26±1.45 | 3.18±1.25 | 0.007* |
| Median (Interquartile Range) | 4.00 (2.00–4.00) | 2.00 (1.00–4.00) |  | 4.00 (2.00–4.00) | 4.00 (2.00–4.00) |  | 2.00 (1.00–4.00) | 4.00 (2.00–4.00) |  |
| Does this nutritional labeling model help me quickly decide which products to buy? | | | | | | | | | |
| Mean ± Standard Deviation | 3.11±1.42 | 3.42±1.64 | 0.398 | 3.11±1.42 | 3.59±1.31 | 0.029* | 3.42±1.64 | 3.59±1.31 | 0.638 |
| Median (Interquartile Range) | 3.00 (2.00–4.00) | 4.00 (2.00–5.00) |  | 3.00 (2.00–4.00) | 4.00 (2.50–5.00) |  | 4.00 (2.00–5.00) | 4.00 (2.50–5.00) |  |
| Does this nutritional labeling model help me decide when to buy or not buy a product? | | | | | | | | | |
| Mean ± Standard Deviation | 3.28±1.45 | 3.37±1.74 | 0.822 | 3.28±1.45 | 3.74±1.34 | 0.043* | 3.37±1.74 | 3.74±1.34 | 0.317 |
| Median (Interquartile Range) | 4.00 (2.00–5.00) | 4.00 (1.00–5.00) |  | 4.00 (2.00–5.00) | 4.00 (2.50-5.00) |  | 4.00 (1.00–5.00) | 4.00 (2.50-5.00) |  |

* P < 0.05; different letters mean P<0.05 BS = Brazilian FoPNL System ; MS = Mexican FoPNL System

ANOVA (post-hoc Tukey test)
